# Neemazal ® as a possible alternative control tool for malaria and African trypanosomiasis?

**DOI:** 10.1186/s13071-016-1538-x

**Published:** 2016-05-04

**Authors:** R. Serge Yerbanga, Jean-Baptiste Rayaisse, Amélie Vantaux, Ernest Salou, Karine Mouline, François Hien, Annette Habluetzel, Roch K. Dabiré, Jean Bosco Ouédraogo, Philippe Solano, Thierry Lefèvre

**Affiliations:** Institut de Recherche en Sciences de la Santé (IRSS), Bobo Dioulasso, Burkina Faso; Centre International de Recherche Développement sur l’Elevage en zone Subhumide (CIRDES), Bobo Dioulasso, Burkina Faso; MIVEGEC (Maladies Infectieuses et Vecteurs: Ecologie, Génétique, Evolution et Contrôle), UMR IRD 224-CNRS 5290-Université de Montpellier, Montpellier, France; University of Camerino, School of Pharmacy, Piazza dei Costantini, 62032 Camerino, MC Italy; INTERTRYP, UMR 177 IRD-CIRAD, Montpellier, France

**Keywords:** *Anopheles coluzzii*, *Glossina palpalis gambiensis*, African trypanosomes, Malaria parasite, Neem, NeemAzal ®, Host-seeking behaviours, Anthropophily, Vector-borne diseases

## Abstract

**Background:**

Research efforts to identify possible alternative control tools for malaria and African trypanosomiasis are needed. One promising approach relies on the use of traditional plant remedies with insecticidal activities.

**Methods:**

In this study, we assessed the effect of blood treated with different doses of NeemAzal ® (NA, neem seed extract) on mosquitoes (*Anopheles coluzzii*) and tsetse flies (*Glossina palpalis gambiensis*) (i) avidity to feed on the treated blood, (ii) longevity, and (iii) behavioural responses to human and calf odours in dual-choice tests. We also gauged NeemAzal ® toxicity in mice.

**Results:**

In *An. coluzzii*, the ingestion of NA in bloodmeals offered by membrane feeding resulted in (i) primary antifeedancy; (ii) decreased longevity; and (iii) reduced response to host odours. In *G. palpalis gambiensis*, NA caused (i) a knock-down effect; (ii) decreased or increased longevity depending on the dose; and (iii) reduced response to host stimuli. In both cases, NA did not affect the anthropophilic rate of activated insects. Overall, the most significant effects were observed with NA treated bloodmeals at a dose of 2000 μg/ml for mosquitoes and 50 μg/ml for tsetse flies. Although no mortality in mice was observed after 14 days of follow-up at oral doses of 3.8, 5.6, 8.4 and 12.7 g/kg, behavioural alterations were noticed at doses above 8 g/kg.

**Conclusion:**

This study revealed promising activity of NA on *A. coluzzii* and *G. palpalis gambiensis* but additional research is needed to assess field efficacy of neem products to be possibly integrated in vector control programmes.

**Electronic supplementary material:**

The online version of this article (doi:10.1186/s13071-016-1538-x) contains supplementary material, which is available to authorized users.

## Background

Vector-borne parasites are responsible for many of the most harmful diseases affecting humans, accounting for more than 17 % of all infectious diseases episodes, and causing about one million deaths annually [1]. Malaria and human African trypanosomiasis are among the most important vector-borne diseases. These diseases are transmitted via bites of *Anopheles* mosquitoes and tsetse flies, respectively. Malaria alone accounts for more than half of the mortality caused by vector-borne diseases with about 450 000 deaths every year, most of them in children under five years of age succumbing to infections by *Plasmodium falciparum* parasites [2]. Although the number of new human African trypanosomiasis cases has dropped by 73 % between 2000 and 2012 with 6314 cases reported in 2013, an estimated 70 million people are still at risk of infection especially in rural populations [3]. In addition, tsetse flies transmit African trypanosomes to livestock and thus have important agronomic and economic relevance. The prevalence of African animal trypanosomiasis remains high in most of sub-Saharan Africa, where it is considered the most important vector-borne livestock disease causing important impact on nutritional security [4].

The control of vector-borne diseases including malaria and African trypanosomiasis relies heavily on the reduction of vector populations. For example, the use of indoor residual spraying and long-lasting insecticidal nets have been shown to be crucial to the successful reductions of malaria incidence [5–7]. However, the evolution of insecticide-resistant mosquito vectors currently threatens the efficacy of these approaches [7, 8]. Likewise, there is a wide range of vector control techniques for reducing the incidence of African human and animal trypanosomiasis including insecticide impregnated traps and targets, visual and odour baits, live traps, insecticide-treated livestock, sequential aerial spraying, and sterile male releases [9–13]. Despite the existence of such effective tools, human and animal trypanosomes are still having an enormous impact on public health and economic development of Sub-Saharan Africa [4]. Hence, there is a need to develop on complementary novel strategies for the control of the parasitic diseases, ideally based on “active ingredients” readily available in these countries such as neem. As part of this effort, a ‘One Health’ approach is increasingly recognized to be key to effective and sustainable control of vector-borne diseases [14], especially for those exploiting the intimate livestock-man interface such as African trypanosomiasis.

One promising approach relies on the use of traditional plant remedies with insecticidal activity. The ingestion of herbal preparations by vertebrate hosts could indeed be an additional tool for managing vector-borne diseases, provided that the vectorial capacity of insects that blood-feed on a treated subject is reduced and that the preparation is not toxic. Among herbal products active against insects and/or against the parasite they transmit, preparations made from the neem tree, *Azadirachta indica*, are of particular interest. Neem trees, native of India, are widespread in tropical and subtropical regions and have various medicinal properties that have been known for more than 2000 years [14].

Because of their wide range of biological activities, neem-based tools may be developed for the control of parasitic agents and their vectors, i.e. against *Plasmodium* spp. and *Anopheles* spp. mosquitoes [15, 16]. In fact, a substantial number of studies conducted with neem extracts, limonoid enriched preparations or purified azadirachtin report ovicidal, larvicidal, adulticidal, anti-feedant, and repellent properties against different mosquito vectors [17–23]. For example, a recent study by a coauthor of the present work showed an impact of NeemAzal ® (a commercial methanol extract from seed kernels of *A. indica* rich in azadirachtin A, the major bioactive compound) on the blood volume intake of *Anopheles stephensi* after repeated feeding on mice treated with the product at an azadirachtin concentration of 60–150 mg/kg [24]. After the 5th blood meal at the highest dosage, mosquitoes’ oviposition was reduced by 50–65 % and egg hatchability by 70 %. [24]. The possible activity of neem extracts on tsetse fly biology has, to our knowledge, never been explored.

Secondly, neem extracts possess antiplasmodial and transmission blocking activities [25–29]. For example, infectious blood-meals from mice treated with NeemAzal ® at an azadirachtin A dose of 50 mg/kg can completely suppress *Plasmodium berghei* development in *Anopheles stephensi*. A complete blockade of mosquito infection was also recently demonstrated in *Anopheles coluzzii* females membrane fed on *P. falciparum* gametocytaemic blood supplemented with NeemAzal ® at an azadirachtin A dose of 70 μg/ml [27].

Finally, recent findings indicate that azadirachtin displays neurotoxic effects and alters the functioning of the central nervous system (CNS) of insects [30–33]. For example, a study on drosophila showed that azadirachtin can interfere with the insect’s CNS via inhibition of excitatory cholinergic transmission and by partly blocking calcium channels [31]. Neurophysiological effects of neem extracts may impact on the vectorial capacity, in particular on the vector's probability to become infected and its capacity to transmit a pathogenic agent. Knowledge of the degree of contacts between humans and disease vectors is crucial to predict the intensity of parasite transmission. The frequency of contacts depends on the vector’s host choice, which is influenced by innate preference. Briefly, odour or visual cues from a potential vertebrate host are encoded in electrical signals in sensory neurons and are eventually processed by the CNS. For example, recent studies showed that mosquito preference for vertebrate hosts is accompanied by changes in the peripheral chemosensory system [34, 35]. Despite the epidemiological importance of the feeding preference, no study has yet explored whether host-seeking behaviour of insect vectors can be altered by exposure to azadirachtin.

Using the mosquito *Anopheles coluzzii*, a major vector of *P. falciparum*, and the tsetse fly *Glossina palpalis gambiensis*, an important vector of African animal and human trypanosomes, the current study determined the effect of blood treated with NeemAzal ® at different doses on (i) the avidity to feed on the treated blood; (ii) longevity of the insects; and (iii) their behavioural responses to human and calf odours in dual-choice tests. Finally, we gauged NeemAzal ® toxicity using mice.

## Methods

### Biological materials

#### Mosquitoes

Laboratory-reared *An. coluzzii* were obtained from an outbred colony established in 2008 and repeatedly replenished with F1 from wild-caught mosquito females collected in Kou Valley (11°23′14"N, 4°24′42"W), situated 30 km from Bobo Dioulasso in south-western Burkina Faso. Mosquitoes were held in 30 × 30 × 30 cm mesh-covered cages at the “Institut de Recherche en Sciences de la Santé” in Bobo Dioulasso under standard conditions (27 ± 2 °C, 70 ± 5 % relative humidity, 12 h:12 h LD rhythm). Females were maintained on rabbit blood and adult males and females fed with 5 % glucose. Larvae were reared at a density of about 300 larvae in plastic trays containing 700 ml of water and were fed with Tetramin® fish food. Three to four day old *An. coluzzii* females were used in the experiments.

#### Tsetse flies

Laboratory-reared *G. p. gambiensis* were obtained from a colony established in 1975 at the “Centre International de Recherche et de Développement sur l’Elevage en zone Subhumide” (CIRDES) in Bobo Dioulasso. Flies were held in 4.5 × 13 × 8 cm mesh-covered cages at a maximum density of 35 flies per cage under stable climatic conditions of 25 ± 2 °C, 70 ± 5 % relative humidity and 12 h:12 h LD rhythm. Flies were fed irradiated bovine blood collected from the local abattoir according to standard rearing procedures using an *in vitro* silicon membrane feeding system [36]. Two day- old teneral *G. p. gambiensis* males were used for the experiments. The choice for males was driven by insectary requirements (logistic rationale), females being needed to maintain the colony. However, both males and females are haematophageous, and do not display any differences in terms of host preferences [37].

#### Neem extract

NeemAzal ® (NA), a commercial extract from neem seed kernels containing 33 %, azadirachtin A 16 % other azadirachtins (B to K), 4 % salannin and 2 % nimbin (Trifolio-M GmbH,Lahnau, Germany, batch number 052) was used. Stock solutions of NA were prepared in DMSO (Dimethyl sulfoxide) at 50 mg/ml. Corresponding volumes of the stock solution were diluted in blood to obtain NA concentrations of 2000 μg/ml, 1000 μg/ml and 500 μg/ml. For experiments related to tsetse flies these concentrations were further diluted by a factor ten and forty.

Separate groups of flies/mosquitoes were used in a series of experiments that systematically evaluated impacts of NA on blood-feeding (b), knock-down (c), survival (d) and behaviour (e).

### Blood-feeding assays

#### Mosquitoes

To determine the impact of NeemAzal ® on *An. coluzzii* blood-feeding success, females were exposed to treated blood-meals using a membrane feeding system and the proportion of fully-engorged females was recorded (Additional file 1: Figure S1a). Mosquitoes were held in paper cups at a density of 80 individuals and each cup was placed under a membrane microfeeder (Additional file 1: Figure S1a). Two paper cups (i.e. 160 mosquitoes) were randomly assigned to each treatment. The experiment included 3 NA treatment groups at different dosages and one control group: (i) NA 500 μg/ml; (ii) NA 1000 μg/ml; (iii) NA 2000 μg/ml; and (iv) control: blood supplemented with an equal amount of DMSO solvent solution. Mosquito females were allowed to feed on their assigned blood treatment for 30 min after that fed and unfed mosquitoes were counted. Three experimental replicates were conducted and a total of 1918 mosquito females were used.

#### Tsetse flies

The impact of NeemAzal ® on fly blood-feeding success was determined by exposing flies to treated blood using a membrane feeding system (Additional file 1: Figure S1b) and measuring repletion of flies. A repletion score of 0 indicates unfed flies while a score of 4 indicates maximum engorgement. Experimental male flies were held in 4.5 × 13 × 8 cm cages at a density of 30 flies and cages placed on a membrane floating in a rack with blood (Additional file 1: Figure S1b). Two cages (i.e. 60 flies) were randomly assigned to NA treated-groups of three different concentrations: (i) NA 500 μg/ml; (ii) NA 1000 μg/ml; (iii) NA 2000 μg/ml; or (iv) to the control group, (which consisted of blood supplemented with an equal amount of solvent solution only). Flies were allowed to feed for 30 min. Two experimental replicates were conducted and a total of 480 flies were used across all four treatments. Unlike mosquitoes, a high proportion of flies were knocked-down following NA-treated meals indicating that these concentrations were likely to be too high to study fly behaviours. We therefore repeated this experiment with doses reduced by a factor of ten: NA 50 μg/ml, NA 100 μg/ml and NA 200 μg/ml (one replicate for each dose and a total of 240 flies across all four treatments) and by a factor of forty: NA 12.5 μg/ml, NA 25 μg/ml and NA50 μg/ml (6 replicates for each dose and a total of 1440 flies across all four treatments).

### Knock-down assays

Following preliminary observations made in the fly blood-feeding assay (b), we measured the effect of different NA concentrations on the proportion of knocked-down (KD) flies (i.e. inability to fly or stand) over time. Cages of 20 flies each were exposed to: (i) NA 500 μg/ml; (ii) NA 1000 μg/ml; (iii) NA 2000 μg/ml; or (iv) a control blood. This procedure was repeated once with the same doses (i.e. 2 replicates with a total of 1120 flies), conducted once with doses reduced by a factor of ten (NA 50 μg/ml, or NA 100 μg/ml, or NA 200 μg/ml, or a control group) and once with doses reduced by a factor of forty (NA 12.5 μg/ml, NA 25 μg/ml, NA 50 μg/ml or a control group). The number of KD flies was counted every day until all KD flies had recovered. Counts were effected starting the day after blood feeding (= day 1) up to day 7.

### Longevity assays

#### Mosquitoes

To determine the impact of NeemAzal ® on mosquito longevity, 40 blood-fed females from each treatment group (control, NA 500 μg/ml, NA 1000 μg/ml and NA 2000 μg/ml) were placed in two 30 × 30 × 30 cm cages (i.e. 2 cages of 20 mosquitoes for each treatment group) and kept under observation for 7 to 18 days. Every morning, dead mosquitoes were counted and removed from the cages until all mosquitoes had died. The experiment was conducted twice under different conditions: 1. without and 2. with supply of cotton pads soaked with glucose solution at 5 %.

#### Tsetse flies

Twenty blood-fed flies from each treatment group (control, NA 500 μg/ml, NA 1000 μg/ml and NA 2000 μg/ml) were placed in a 4.5 × 13 × 8 cm cage with no access to blood. Every morning dead flies were counted and removed from the cages. Two replicates were run and a total of 160 flies were used. We repeated this experiment with NA doses reduced by a factor of ten (one replicate and a total of 80 flies) and NA doses reduced by a factor of 40 (6 replicates and a total of 480 flies). Survival was monitored until all individuals died.

### Behavioural assays

#### Mosquitoes

To determine the impact of NA on mosquito host-seeking behaviour, the responses of mosquitoes to human and calf odours was studied, using a dual-port olfactometer. The experiments were conducted with females three days after the ingestion of NA treated blood-meals (NA 500 μg/ml, NA 1000 μg/ml, NA 2000 μg/ml, and controls). During these three days, mosquitoes were held in 30 × 30 × 30 cm cages with access to a 5 % glucose solution and provided with Petri dishes covered with wet filter paper for oviposition. The olfactometer and the general procedures adopted were similar to those described previously [38]. Odour stimuli came from two tents connected to two collecting boxes of the olfactometer by air vent hoses (Scanpart®) (Additional file 1: Figure S2). Fans, setup at the junction of the air vent hose and the tent, drew air from the tents to the olfactometer, providing the odour laden air current against which mosquitoes were induced to fly. Mosquito netting was placed at the junction of the air vent hose with the collecting boxes to restrain responding mosquitoes inside the box. The tents were located outdoors and the olfactometer inside an experimental room. The air speed in the release cage of the dual-port olfactometer was regulated at 15 cm⁄ s (±2 cm/s) using a Testo 425- Compact Thermal Anemometer (Testo, Forbach, France) equipped with a hot wire probe [range: 0 to + 20 m⁄ s, accuracy: ±(0.03 m⁄ s + 5 % of mv)]. The temperature in the experimental room was 27.5 ± 2.5 °C and the relative humidity 80 ± 10 %.

In the morning of the experimental day, mosquitoes were placed in paper cups with access to water only. Mosquitoes were marked with one of three different coloured powders (Luminous Powder Kit, BioQuip) corresponding to their treatment group. The matching between blood treatment and colours was switched between each test. In the evening, about 20 mosquitoes from each treatment (except for mosquitoes from the NA 2000 dose which displayed a very low survival rate) were simultaneously placed into the releasing cage and allowed to respond for 30 min. During this time, mosquitoes that were activated by the stimuli left the releasing cage and flew upwind into the collecting boxes from which they were retrieved. Three tests per evening were carried out between 7 pm and 10 pm. Odour stimuli (human *vs* calf odour) were alternated between the right and left arm to avoid any position effect. Six combinations of human volunteers (*n* = 4) and calves (*n* = 3) were used as odour sources to obviate any effects related to the individual volunteer or calf. All volunteers who served as a human odour source were male residents of Bobo Dioulasso, between 21 and 35 years old and weighed 60–74 kg. Volunteers were in good health. Before the test, volunteers were asked not to take a shower, not apply any perfume or repellent, not to smoke or eat garlic and onion [39, 40]. The volunteers acting as odour sources sat on a chair inside the tent. For calf odour stimuli, we used zebus of similar mass as that of human volunteers.

Each batch of mosquitoes was tested once. Nine tests were conducted with a total of 177 controls, 175 NA500 μg/ml, 123 NA1000 μg/ml and 28 NA2000 μg/ml treated mosquitoes released. At the end of each test, the mosquitoes inside the two upwind collecting boxes and the releasing box were removed with an aspirator and counted. Two behavioural responses were gauged: *activation rate* = proportion of mosquitoes caught in both collecting boxes out of the total number released, that is the proportion of mosquitoes engaging in take-off and up-wind flight; and *anthropophilic rate* = proportion of mosquitoes caught in the collecting box emitting human odour out of the total number retrieved from both collecting boxes, that is the proportion of mosquitoes flying towards human volunteers’ odours. These two behavioral traits are part of the sequence leading a mosquito towards its host, and through which host-seeking is expressed.

#### Tsetse flies

To determine the impact of NA on tsetse host-seeking behaviour, we studied the responses of tsetse flies to human and calf stimuli using a flight chamber. Flies of 4 treatment groups were used two days after the ingestion of experimental blood-meals: NA 12.5 μg/ml, NA 25 μg/ml, NA 50 μg/ml, and a control group. Unlike the dual-port olfactometer, which measures odour-mediated behavioural responses, the chamber device allows the assessment of both long- and short-range stimuli and includes olfactory stimuli, visual cues, warmth, moist convective currents and host movement. The flight chamber consisted of two choice rooms occupied either by a human or a calf host of two rooms separated by a curtain (Additional file 1: Figure S3) and a narrow central box used to release the flies. The day prior to testing, flies were marked with acrylic paint (OIL18, Royal & Langnickel, Royal brush Mfg Ltd, UK) on the pronotum with one of three colours according to their treatment group. Dual-choice tests were conducted between 10 am and 1 pm. Between 17 and 24 flies from each blood treatment were simultaneously released in the central chamber. Following a twenty minutes acclimatization period, drapes were opened, allowing access to the vertebrate hosts. Dual-choice tests were conducted for 30 min, after which drapes were closed and the number of flies in each chamber counted. Host stimuli (human *vs* calf) were alternated between the right and left host chamber to avoid any position effect. Five combinations of human volunteers (*n* = 2) and calves (*n* = 3) were used as stimuli sources to minimize effects of individuals. The volunteers acting as odour sources were standing in the chamber and equipped with a net to catch approaching and landing insects. For calf odour stimuli, we used zebus as illustrated above. The calf was anchored loosely with a rope to prevent it from escaping but not from moving.

Each batch of flies was tested once and overall 16 tests were conducted with a total of 280 controls, 278 NA12.5, 307 NA25 and 313 NA50 treated flies. As in the mosquito behavioural test, two behavioural responses were gauged: *activation rate* = proportion of flies caught in both host chambers out of the total number released; and *anthropophilic rate* = proportion of flies caught in the host chamber emitting human stimuli out of the total number retrieved from both host chambers.

### *In vivo* toxicity assay

The acute toxicity test was performed in mice according to the method of Thompson and Weil [41]. Experiments were carried out in accordance with the suggested ethical guidelines for care of laboratory animals by the Act n°00468 promulgated in January 24th, 1994 that covered all West African French Speaking Countries. The mice NMRI (Naval Medical Research Institute) were obtained from the “Centre International de Recherche et de Développement sur l’Elevage en Zone Subhumide (CIRDES)” of Bobo-Dioulasso. They were maintained under standard conditions (Temperature 25 °C, light 12 h, free access to water) in cages in groups of three and starved for 24 h before the experiment. Four different dosages starting from a 2500 mg/kg of stock solution and increasing by a factor 1.5 were tested: 3750 mg/kg; 5625 mg/kg; 8437.5 mg/kg; and 12,656.25 mg/kg. Groups of three 8–12 weeks old male and female NMRI mice were administered a single oral treatment of the extracts at each dosage; control mice received only the solvent. Animals were kept under observation for 48 h and any signs of toxicity were recorded. Body weight data were taken on day 0 (before extract administration) and day 14 post-treatment.

### Statistical analysis

All statistical analyses were performed in R (version 2.15.3). A logistic regression by Generalized Linear Mixed Model (GLMM, binomial errors, logit link; lme4 package) was used to investigate the effect of NA treatment on mosquito feeding rate. In this model, NA treatment was coded as a fixed effect and replicate number as a random effect. A GLMM with Poisson errors was used to explore the effect of NA treatment (fixed effect) on tsetse fly repletion score with replicate as a random effect. The effect of NA treatment on the proportion of KD flies was analyzed using a GLMM with binomial error. Time post blood meal was included as a covariate. We also included cage identity as a random factor to account for the repeated measurements overtime (i.e. pseudoreplications). The effect of NA treatment on mosquito survivorship was analysed using Cox’s proportional hazard mixed regression models (“coxme” function in the “coxme” package [42]). Because all the mosquitoes from the first survivorship experiment (in deprived sugar conditions, see above) died eventually, there was no censoring in this case. In contrast, since individuals were still alive when last seen on day 18 post-treatment, the analysis of the survival data from the second experiment (when mosquitoes were allowed to feed on a 5 % glucose solution) accounted for censoring. NA treatment was specified as a fixed effect and cage number as a random effect. Cox’s mixed models were also used to analyse tsetste fly survival data with treatments specified as a fixed effect and replicate as a random effect. Binomial GLMMs were fitted to investigate mosquito and tsetse fly activation and anthropophilic rates. In these GLMMs, NA treatment was coded as a fixed factor, while test number and host combination were coded as random factors. In these GLMMs, the individual insect was used as a statistical unit. Given that mosquitoes/flies in a trial were not independent (pseudoreplications), this means that the magnitudes of our confidence intervals were underestimated. Finally, a GLMM with a Gaussian distribution was used to explore the effect of NA treatment (5 doses: 0; 3750; 5625; 8437.5; and 12,656.25 mg/kg), time (2 levels: before *vs* 14 days after treatment) and mouse sex (male *vs* female) on mouse weight. Mouse identity was coded as a random effect to account for pseudoreplications.

Model simplification used stepwise removal of terms, followed by likelihood ratio tests (LRT). Term removals that significantly reduced explanatory power (*P* < 0.05) were retained in the minimal adequate model [43]. When the main effect of NA treatment was statistically significant based on the LRT test, *post-hoc* multiple pairwise comparisons were run using the “glht” function of the “multcomp” package.

### Ethical statement

Ethical approval was obtained from the Centre Muraz Institutional Ethics Committee under agreement no.A003- 2012/CE-CM. The protocol conforms to the declaration of Helsinki on ethical principles for medical research involving human subjects (version 2002) and informed written consent were obtained from all volunteers.

## Results

### Effects on blood-feeding

**Fig. 1:**
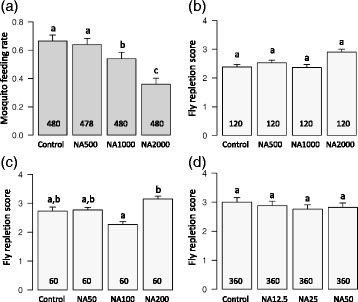
Effect of different doses of NeemAzal ® on *An. coluzzii* and *G. p. gambiensis* fly blood-feeding. **a** *An. coluzzii* feeding rates, expressed as the proportion (+95 % CI) of engorged mosquitoes out of the total number exposed to the blood-meal (3 experimental replicates were conducted involving a total of 1918 mosquito females). Mosquitoes received blood supplemented with NeemAzal ® at doses of 0 μg/ml (i.e. control group consisting of blood supplemented with DMSO solvent only), 500 μg/ml (NA500), 1000 μg/ml (NA1000) and 2000 μg/ml (NA2000). **b** Mean repletion score (0 to 4) (+ SEM) of tsetse flies exposed to NeemAzal ® concentrations similar to that of mosquitoes (2 experimental replicates, in total 480 flies were examined). **c** Mean repletion score (0 to 4) of flies exposed to NeemAzal ® concentrations reduced by a factor of ten (1 experimental replicate, in total 240 were flies examined). **d** Mean repletion score (0 to 4) of flies exposed to NeemAzal® concentrations reduced by a factor of 40 (6 experimental replicates, totally 1440 flies examined). Numbers in bars indicate the sample size for each dose. Different letters above the bars denote statistically significant differences based on *post-hoc* multiple pair-wise comparisons

NeemAzal reduced feeding rates of mosquitoes in a concentration dependent manner (*X*^2^ = 118.71, *df* = 3, *P* < 0.0001, Fig. 1a). At the highest dosage of 2000 μg/ml, only about 1/3 of females (36 % ± 4.3) took a blood meal compared to about 2/3 (67 % ± 4.2) in controls. About half of the mosquitoes fed on blood treated with NA at 1000 μg/ml (54 % ± 4.5), corresponding to a reduction of feeding of about 20 % compared to the controls. No difference in feeding rates was observed between NA500 and control females (Fig. 1a).

#### Tsetse flies

In the first set of NA doses (i.e. concentrations similar to that used for mosquitoes), very few flies remained unfed (13 out of 480) and the mean repletion score was 2.55 ± 0.05. There was a marginally significant effect of NA treatment (*X*^2^ = 8.5, *df* = 3, *P* = 0.04). Flies fed on blood supplemented with a concentration of 2000 μg/ml of NA displayed slightly higher scores than flies fed on the other NA treatments (although *post-hoc* multiple comparison tests indicated that these differences were not significant, Fig. 1b; Control: 2.38 ± 0.09, NA500: 2.53 ± 0.09, NA1000: 2.37 ± 0.1, NA2000: 2.9 ± 0.1). In the second set of NA doses (concentrations reduced by a factor of ten) only 2 flies out of 240 remained unfed and the mean repletion score was 2.73 ± 0.056. There was a significant effect of NA treatment (*X*^2^ = 8.7, *df* = 3, *P* = 0.033) with higher repletion scores at NA 200 μg/ml compared to 100 μg/ml (Fig. 1c; Control: 2.73 ± 0.14, NA50: 2.77 ± 0.09, NA100: 2.27 ± 0.095, NA200: 3.15 ± 0.1). Finally in the third set of NA doses (concentrations reduced by a factor of 40), 63 flies out of 1440 remained unfed and the mean repletion score was 2.87 ± 0.03. At these doses, there was no effect of NA treatment on fly repletion score (*X*^2^ = 4.2, *df* = 3, *P* = 0.24; Fig. 1d, Control: 3 ± 0.16, NA50: 2.88 ± 0.15, NA100: 2.76 ± 0.15, NA200: 2.82 ± 0.15).

### Knock-down effects on flies

**Fig. 2:**
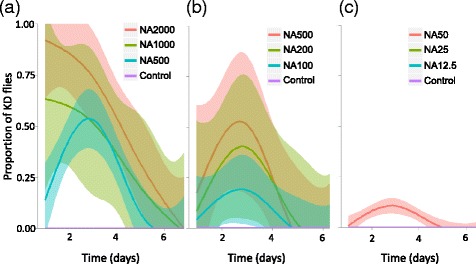
Knock-Down (KD) effect of different doses of NeemAzal ® on *G. p. gambiensis*. **a** Proportion of knocked-down flies over 7 days of observation (day 1 = day after blood meal). Flies received blood supplemented with NeemAzal ® doses of 0 μg/ml (control group consisting of blood supplemented with DMSO solvent solution only), 500 μg/ml (NA500), 1000 μg/ml (NA1000) and 2000 μg/ml (NA2000) (2 replicates, in total 1120 were flies examined). **b** Proportion of KD flies over time with NeemAzal ® concentrations reduced by a factor of 10 (1 replicate, totally 560 flies examined). **c** Proportion of KD flies over time with NeemAzal ® concentrations reduced by a factor of 40 (1 replicate, in total 560 flies were examined). Each colour line represents a LOESS curve fitted to each treatment group

NA-treated blood meals induced knock-down in tsetse flies but not in *Anopheles* mosquitoes as observed from preliminary studies. Experiments conducted with flies revealed that the proportion of KD flies was strongly affected by the NA concentrations (first set of NA doses: *X*^2^ = 251, *df* = 3, *P* < 0.0001, Fig. 2a; second set of NA doses: *X*^2^ = 38, *df* = *P* < 0.0001, Fig. 2b; third set of NA doses: *X*^2^ = 12.7, *df* = 3, *P* = 0.005, Fig. 2c). At the highest dosage (2000 μg/ml) an immediate knock-down of about 90 % of flies was recorded, whereas at 500 μg/ml a maximal KD rate of about 50 % was observed on day 3. For each set of doses, KD flies recovered some flying and walking abilities after a period of time (time effect, first set of NA doses: *X*^2^ = 241, *df* = 1, *P* < 0.0001; second set of NA doses: *X*^2^ = 23, *df* = 1, *P* < 0.0001; third set of NA doses: *X*^2^ = 6, *df* = 1, *P* = 0.01). Recovery of KD flies was observed starting day 5 by flies treated with 100 to 500 μg/ml and at day 7 by those belonging to the 1000 and 2000 μg/ml treatment group.

For the highest set of doses (i.e. NA 500, 1000 and 2000 μg/ml), there was also a significant time by treatment interaction (*X*^2^ = 20, *df* = 3, *P* = 0.0002) such that the temporal dynamics of fly recovery was different among the treatments. In particular, while the proportion of KD flies that fed on blood treated with NA doses of 2000 and 1000 μg/ml was maximal on the first day post treatment, this proportion peaked on day three post-treatment for lower doses (i.e. NA500, NA200, NA100 and NA50). No KD effect was observed for controls as well as NA 12.5 and NA 25 treatments (Fig. 2).

### Effects on longevity

#### Mosquitoes

**Fig. 3:**
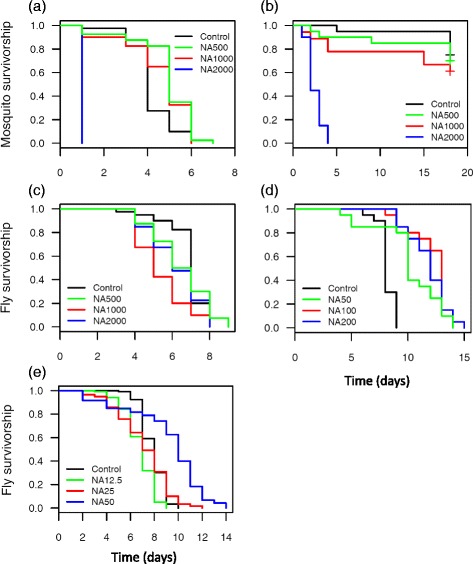
Effect of different doses of NeemAzal ® on *An. coluzzii* and *G. p. gambiensis* survivorship. **a** *An. coluzzii* survivorship in sugar-deprived condition. Mosquitoes received blood supplemented with NeemAzal ® doses of 0 μg/ml (blood supplemented with solvent solution only), 500 μg/ml (NA500), 1000 μg/ml (NA1000) and 2000 μg/ml (NA2000) (*n* = 160 mosquitoes). **b** Mosquito survivorship when provided *ad libitum* 5 % glucose solution (*n* = 80 mosquitoes). **c** Survivorship of flies exposed to NeemAzal ® at concentrations similar to those given to mosquitoes (*n* = 160 flies). **d** Survivorship of flies exposed to NeemAzal ® concentrations reduced by a factor of 10 (*n* = 80). **e** Survivorship of flies exposed to NeemAzal ® concentrations reduced by a factor of 40 (*n* = 480)

Mosquito survival was found to be influenced by NA treatment under both alimentary post blood meal conditions, namely deprived (*X*^2^ = 139, *df* = 3, *P* < 0.0001, Fig. 3a) *versus* provided *ad libitum* with sugar solution (*X*^2^ = 57, *df* = 3, *P* < 0.0001, Fig. 3b). In stressful conditions (i.e. no access to sugar solution) all mosquitoes from the NA2000 died by day 1 *vs* day 6 or 7 for the other group (Table 1, Fig. 3a). Providing mosquitoes with a source of sugar post treatment significantly improved their survival. As in the experiment measuring survival impact under stressful alimentary conditions, NA given at 2000 μg/ml exerted a strong toxic effect, reducing mean survival to 2.65 days and killing all mosquitoes of that group by day 4 (Table 1, Fig. 3b).

**Table 1:** Risk of mortality (hazard ratio) along with the 95 % CI, *z* and *P*-value for each treatment group relative to the control

Assay	Treatment	Hazard ratio (lower .95 - upper .95)	*z*	*P*-value
Mosquito survival assay 1				
	NA500	0.43 (0.27–0.68)	3.6	0.0002
	NA1000	0.52 (0.33–0.82)	2.8	0.005
	NA2000	30.54 (13.13–71.05)	7.9	2.11e^-15^
Mosquito survival assay 2				
	NA500	1.31 (0.4–4.3)	0.447	0.6
	NA1000	2 (0.6–6)	1.139	0.2
	NA2000	40 (11–140)	5.773	7.8e^-9^
Fly survival assay 1				
	NA500	0.9 (0.57–1.4)	0.404	0.68
	NA1000	2.17 (1.39–3.37)	3.418	0.0006
	NA2000	1.24 (0.8–1.9)	0.969	0.33
Fly survival assay 2				
	NA50	0.08 (0.03–0.19)	5.5	3.17e^-08^
	NA100	0.04 (0.02–0.12)	6.5	8.13e^-11^
	NA200	0.05 (0.02-0.13)	6.4	1.82e^-10^
Fly survival assay 3				
	NA12.5	1.2 (1.05–1.7)	1.1	0.06
	NA25	0.93 (0.7–1.2)	0.5	0.56
	NA50	0.25 (0.18–0.33)	9	< 2e^-16^

#### Tsetse flies

In the first set of tested NA doses (i.e. NA 500, NA 1000, NA 2000 μg/ml), fly survival was slightly but significantly affected by treatment (*X*^2^ = 15.7, *df* = 3, *P* = 0.0013, Fig. 3c). Control flies survived significantly better than flies fed on blood supplemented with NA 1000 μg/ml Table 1). In the second and third set of NA doses (i.e. NA concentrations reduced by a factor of ten and 40 respectively), NA treatment was found to exert a contrary effect, i.e. to prolong survival (*X*^2^ = 51, *df* = 3, *P* < 0.0001 and *X*^2^ = 187, *df* = 3, *P* < 0.0001, Fig. 3c, d). In the second set, control flies were all found to be dead at day 9 compared to treated flies on day 14 to 15 (Fig. 3c, Table 1). In the third set, NA50 flies showed a slightly increased survival compared to controls as in the second set experiment, whereas the mean longevity of flies treated with NA12.5 and NA25 was not different from that of the controls (Fig. 3c, Table 1).

In summary, NA was found to exhibit a negative effect on fly longevity at high concentrations (500, 1000 and 2000 μg/ml), a positive effect at intermediate concentrations (i.e. 50, 100 and 200 μg/ml), and no effect at low concentrations (12.5 and 25 μg/ml).

### Effects on behaviour

#### Mosquitoes

**Fig. 4:**
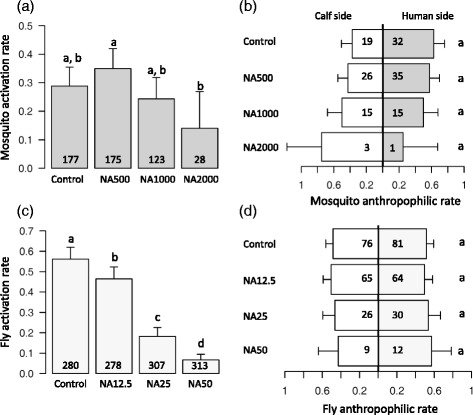
Effect of different doses of NeemAzal ® on *An. coluzzii* and *G. p. gambiensis* host-seeking behaviour. **a** *An. coluzzii* activation rate, expressed as the proportion of mosquitoes caught in both collecting boxes out of the total number released in the downwind cage (*n* = 503 mosquitoes). **b** *An. coluzzii* anthropophilic rate, expressed as the proportion of mosquitoes caught in the collecting box emitting human odour out of the total number retrieved from both collecting boxes (*n* = 146). Mosquito behaviour was gauged three days after receiving blood supplemented with NeemAzal ® doses of 0 μg/ml (control group consisting of blood supplemented with solvent solution only), 500 μg/ml (NA500), 1000 μg/ml (NA1000) and 2000 μg/ml (NA2000). Dar grey bars: human side, white bars: calf side (**c**) *G. p. gambiensis* activation rate, expressed as the proportion of flies caught in both host chambers out of the total number released in the central chamber (*n* = 1178 flies), **d** *G. p. gambiensis* anthropophilic rate, expressed as the proportion of fly caught in the human chamber out of the total number retrieved from both host chambers (*n* = 363). Fly behaviour was gauged two days after receiving NeemAzal ® doses of 0 μg/ml (control), 12.5 μg/ml (NA12.5), 25 μg/ml (NA25) and 50 μg/ml (NA50). Light grey bars: human side, white bars: calf side

There was a marginally significant effect of NA treatment on mosquito activation rate (*X*^2^ = 8, *df* = 3, *P* = 0.04, Fig. 4a). Of the 177 untreated mosquitoes released in the downwind cage, 51 flew upwind into one of the two collecting boxes, corresponding to an activation rate of 28.8 ± 6.7 %. The activation rate of mosquitoes fed on blood supplemented with NA at a concentration of 2000 μg/ml appeared to be reduced to half (14 ± 6.7 %), however, *post-hoc* multiple pairwise comparison tests indicated that this difference was not statistically significant (Fig. 4a) due to the small number of examined individuals. In fact, the high mortality of mosquitoes fed on NA2000 μg/ml blood (Fig. 3a, b) allowed only 28 individuals from this group to be tested. An acceptable number of mosquitoes could be observed in the NA1000 μg/ml group (*n* = 123), however, the recorded activation rate of 24.3 ± 7.6 %, similar to that of the controls, indicated no NA impact even at this relatively high dosage. NA treatment had no detectable effect on mosquito odour-mediated preference (*X*^2^ = 3, *df* = 3, *P* = 0.4, Fig. 4b). Mosquitoes from the control group displayed a similar degree of anthropophily as those from treatment groups (i.e. departure from a 50–50 distribution, *z*- value = 1.8, *P* = 0.0719). An anthropophilic rate of 62.7 ± 13 % was measured in control mosquitoes, compared to 57.4 ± 12.4 % in NA500 μg/ml mosquitoes, 50 ± 18 % in NA1000 μg/ml mosquitoes and 25 ± 42 % in NA2000 μg/ml mosquitoes (Fig. 4b).

#### Tsetse flies

Of the 280 untreated *G. p. gambiensis* released in the central chamber, 157 flew into one of the two host chambers indicating an activation rate of 56 ± 5.8 %. In NA treated flies, a strong impact on fly activation emerged (*X*^2^ = 278, *df* = 3, *P* < 0.0001, Fig. 4c). Fly activation rates were found to steadily decrease with increasing concentrations of NA in the dose range 12.5–50 μg/ml (Fig. 4c, all multiple pairwise comparisons tests were significant). Fly activation rates ranged from as little as 6.7 ± 2.8 % for NA50 flies to 18.2 ± 4.3 % in NA25 flies, 46.4 ± 5.9 % in NA12.5 flies and 56 ± 5.8 % in control flies.

There was no effect of NA treatment on fly attraction toward human or calf stimuli (*X*^2^ = 0.8, *df* = 3, *P* = 0.85, Fig. 4d) and control flies did not display any preference for either host stimuli (i.e. departure from a 50–50 distribution, *z*- value = 0.66, *P* = 0.511). Fly anthropophilic rates amounted to 51.6 ± 7.8 % in control flies, 49.6 ± 8.6 % in NA12.5 flies, 53.6 ± 13 in NA25 flies and 57.1 ± 21 % in NA 50 flies (Fig. 4d).

### *In vivo* toxicity

At the tested doses, namely 3750 mg/kg, 5625 mg/kg, 8437.5 mg/kg and 12,656.25 mg/kg no mortality was observed after 14 days of follow-up. However, at oral doses above 8 g/kg, alterations in behaviour of the mice, namely hyper activity for about thirty minutes and piloerection were noticed.

**Fig. 5:**
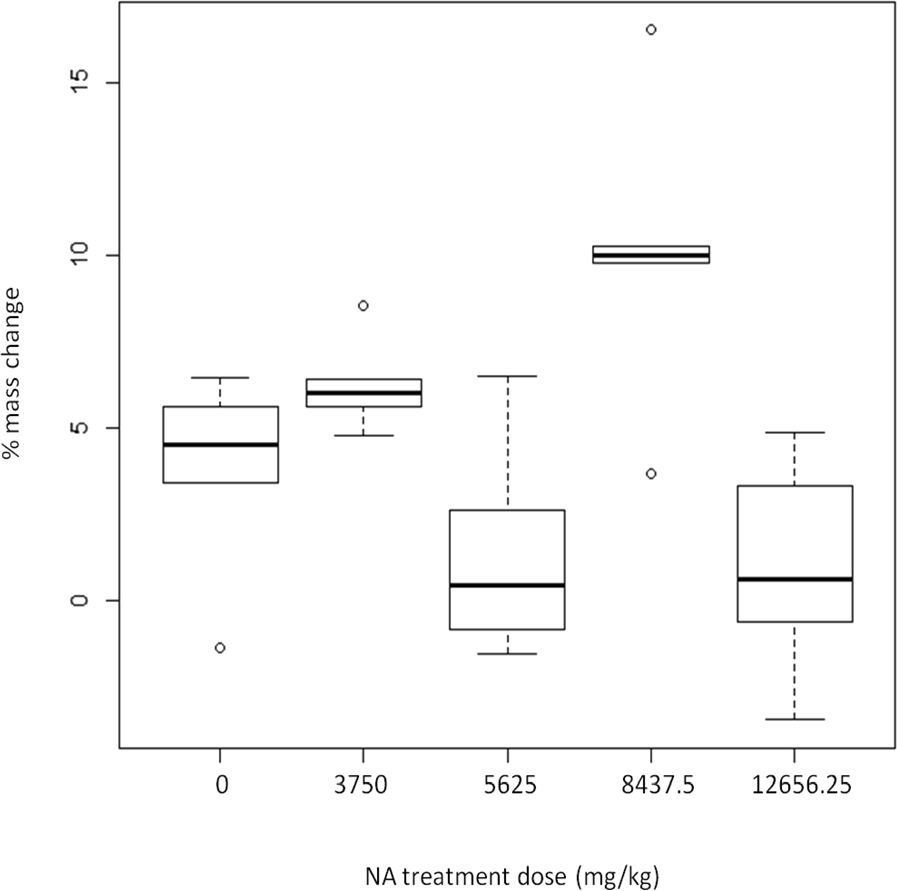
Percentage mass change of mice between day 0 (before ingestion of one of 4 doses of NA) and day 14. Six mice (3 females and 3 males) were used in each treatment group: NA 3750 mg/kg, 5625 mg/kg, 8437.5 mg/kg, and 12,656.25 mg/kg), controls received blood supplemented with solvent solution only). The box plots indicate the median (large horizontal bars), the 25th and 75th percentiles (squares), the minimum and maximum values (whiskers) and outliers (circles)

Overall, mice gained weight over the 14 day period after the ingestion of NA across all treatment doses (*X*^2^ = 22, *df* = 1, *P* < 0.0001, Fig. 5). However, mass gain was significantly influenced by NA treatment (dose by time interaction: *X*^2^ = 27, *df* = 4, *P* < 0.0001, Fig. 5). There was no sex effect (*X*^2^ = 0.13, *df* = 1, *P* = 0.71), no sex by time interaction (*X*^2^ = 1.53, *df* = 1, *P* = 0.22), and no three-way interactions between sex, time and dose (*X*^2^ = 7.53, *df* = 4, *P* = 0.11).

## Discussion

Plant-derived chemicals may provide novel effective tools against vector-borne diseases. We evaluated the effects of a single blood-meal supplemented with different concentrations of NeemAzal ® on the blood-feeding success, survival, and anthropophily - measured here as response to host-derived stimuli - of *An. coluzzii* and *G. palpalis gambiensis*.

Mosquitoes offered blood-meals treated with doses equal or superior to 1000 μg/ml of NA by membrane feeding were less likely to engorge than mosquitoes exposed to control blood or blood treated with 500 μg/ml of NA. This result confirms the existence of a primary antifeedant effect of neem products in *Anopheles* mosquitoes as reported for other insect species even though in this study the effect was observed only at a very high dosage [44]. Similar experiments conducted by Lucantoni et al. [18] with *An. stephensi* showed that only 50 % of females offered NA treated meals at 3000 ug/ml (corresponding to an azadirachtin A concentration of 1000 μg/ml, given that the azadirachtin A content of NA is about 30 %) took a blood meal whereas those offered NA at 30 ug/ml (10 μg/ml azadirachtin A) or 300 ug/ml (30 μg/ml azadirachtin A) fed almost all (90 %) as controls. Our results complement that study to suggest that *Anopheles* mosquitoes may possess a sensory apparatus (e.g. chemo-receptors on mouthparts) that allows them to detect potentially toxic compounds in alimentary sources. Unlike mosquitoes, tsetse flies readily fed on NeemAzal ®-treated blood-meals, even at 2000 μg/ml corresponding to 656 μg/ml of azadirachtin A. Because tsetse flies are exclusively hematophageous insects (but see [45]) and given the fact that blood is not a risky source in terms of presence of toxic components, it is plausible that *Glossina* flies have not evolved chemo-receptors for scrutinizing potentially toxic elements in their alimentary source. In this study NA impact on vector feeding behaviour was evaluated after a single administration of the neem product. For comparison, recent studies with *An stephensi* showed an increase of antifeedant effects after multiple blood meals on NA treated mice. At the dosages of 60 and 105 mg/kg, feeding was reduced by 50 to 80 % after the fifth blood meal. Taken together, it appears that NA may interfere with feeding through different mechanisms, involving the sensory apparatus and physiological processes determining readiness to feed [24].

A significant result of our study was the observation of knock-down effects in tsetse flies following the ingestion of blood-meals treated with NA at doses equal or superior to 50 μg/ml. This effect was dose-dependent and reversible: the proportion of KD flies and the duration of paralysis increased with increasing NA concentrations in the blood-meal. At 1000 and 2000 μg/ml the effect was strongest on day one and lasted until day 7 post-blood meal. At doses comprised between 50 and 500 μg/ml of NA, the effect was slowly increasing during the first two days, peaked on day 3 and dropped to zero on day 5 post-blood meal (Fig. 2). The fact that KD flies were able to recover after a few days suggests that NA components responsible for the effect were metabolized by the flies. The proportion of knocked-down flies was strongly NA dose dependent, however, even at the two highest dosages some individual flies were not affected; at 2000 μg/ml still 10 % and at 1000 μg/ml 35 % of flies remained active upon the ingestion of the treated blood-meal. This individual variation in paralysis may result from a fly to fly variation in blood-meal size: unaffected flies might have been those having engorged only a small amount of blood. Unfortunately, we did not concomitantly measure knock-down and blood meal size in the same flies and the question therefore remains open. Finally, care must be taken when interpreting data from the second and third experiment examining effects at dosages ranging from 12.5 to 500 μg/ml, since only one replicate was performed (each using 560 flies, Fig. 2b, c).

In a study testing the insecticidal effect of ivermectin-treated bloodmeals, Langley and Roe [46] observed that tsetse flies dying within 48 h after ingestion of ivermectin initially became lethargic and displayed progressive paralysis of locomotor muscles. It was postulated that such effects were due either to stimulation of presynaptic GABA (a neurotransmitter known to have inhibitory effects on muscular activity) release or to enhanced GABA binding at its receptor sites. Similar mechanisms might be involved in NA-mediated paralysis. One striking difference with this previous study, however, was that our flies recovered and lived for a few days following a paralysis episode. Compared to control flies, high doses of NA (500, 1000 and 2000 μg/ml) induced only a slight negative effect on longevity while a positive effect was observed at intermediate doses (i.e. 50, 100 and 200 μg/ml), and no effect at low doses (12.5 and 25 μg/ml). We speculate that the increased fly longevity at intermediate concentrations could be due to paralysis. In particular, the rate of living theory postulates that the faster an organism’s metabolism, the shorter its lifespan [47]. Accordingly, NA-mediated paralysis may have caused a decreased metabolic rate, which in turn enhanced fly longevity. At high NA concentrations, flies were paralysed too and their metabolic rates were presumably reduced. However, at such doses, the possible positive effect of a reduced metabolic rate on longevity may have been outweighed by the direct negative effect of NA’s insecticidal activity. Looking at the KD results from a field perspective, it can be assumed that knocked-down flies remaining paralysed for 5–6 days are very likely to get predated by insectivores or to die due to exposure to climatic adverse events such as direct sun. From this point of view, the fact that some KD effect was observed with NA at a concentration as low as 50 μg/ml is encouraging, a systemic ectocidal formulation based on an azadirachtin A enriched product may have a significant impact on survival of fly populations.

Following the ingestion of a NA-treated bloodmeal, mosquitoes did not display any conspicuous behavioural alterations including reduced locomotor activity, or knock-down effects such as those observed when exposed to pyrethroids or ivermectin [48]. However, findings by one of the co-authors of the current work indicate that KD effects can occur in *An. stephensi* mosquitoes when feeding on blood spiked with NA at an azadirachtin A concentration ≥ 400 μg/ml (Lucantoni and Habluetzel, unpublished results).

The insecticidal effect of the highest dose of NA (2000 μg/ml) on mosquito longevity was much stronger than in tsetse flies and all mosquitoes were killed by 2.65 days on average. In contrast, mosquito females fed on blood treated with 500 and 1000 μg/ml of NA and given *ad libitum* access to 5 % glucose solution lived up to an estimated 30.3 days and 24.86 days respectively, similar to controls (34.8 days), hence suggesting no impact of low doses of NA on mosquito survival. However, sample sizes used here were relatively small. Thus we cannot rule out that these lower doses of NA had moderate impacts on mosquito survival that could have been undetected here. Longevity is a core component of malaria and trypanosomiasis epidemiology: the longer the vectors live, the more the parasites can be transmitted [49]. For example, *Plasmodium falciparum* requires 10–16 days (according to the temperature) to complete its sporogonic development in the mosquito. After this period, the parasite can be transmitted during all subsequent blood-meals of the mosquito. Regardless of the other bioactive properties of neem (e.g. transmission blocking [27]), our results suggest that a malaria control approach based on the reduction of mosquito longevity using a neem-based phytomedicine would require a high concentration of active components in the blood. Although an important number of studies have been devoted to neem effects on a series of malaria mosquito traits (larval development, reproduction, oviposition behaviour, egg viability, parasite sporogony, reviewed in [44]), very few have focused on adult longevity. This is surprising given the epidemiological importance of this trait [49]. The only study we are aware of reports that the longevity of *Culex tarsalis* and *Culex quinquefasciatus* adult females feeding continuously on 10 and 50 μg/ml of azadirachtin in 10 % sucrose solution after emergence was decreased [20]. Dembo and colleagues [24] mentioned in the discussion section (“data not shown”) not to have found any evidence of NA impacting on *An stephensi* survival even after repeated NA feeding of females on treated mice. Thus, taken together from the results of the NA studies conducted up to the present, it appears that the neem product is likely to impact on mosquito survival - if given as a single treatment - only at a relatively high dosage of 2000 μg/ml (corresponding to an azadirachtin A dose of about 667 μg/ml).

We found that the activation rate of both vectors exposed to host stimuli three days post-bloodmeal was affected by NA. In tsetse flies this effect was clearly dose-dependent: the higher the concentration of NA in bloodmeals, the lesser vectors engaged in host-seeking. Tsetse flies that had fed on blood supplemented with 50 μg/ml of NA displayed an 8 times decrease in activation compared to controls. Mosquitoes having fed on NA 2000 displayed an activation rate of about half of that of the controls. However, no effect of NA was found on anthropophilic rates of experimental mosquitoes and flies. In mosquitoes, there might be failure to detect any significant differences due the low sample size (Fig. 4b). Bloodsucking insects have a well-developed sensorial apparatus to locate and identify specific hosts. The host-seeking process can be divided into a series of behavioural stages, which show considerable variation among vector species [50]. First, mosquitoes and tsetse flies generally engage in a non-oriented, random flight called kinesis (or appetitive search) that aims at maximising the probability of contact with host-derived stimuli (this is what we have coined *activation rate,* i.e. the proportion of insects that left their releasing cage to fly toward host stimuli) [51–54]. In the absence of host stimuli, mosquitoes usually fly upwind in search of an odour plume from a potential vertebrate host. This behaviour can be triggered and modulated by both endo- and exogenous factors including circadian rhythm, nutritional condition, development, CO2 and odours [55, 56]. Upon detection of host cues, the insect then switches to directional orientation towards the stimulus to locate the host (i.e. long range positive taxis). At this stage, if cues from several potential blood sources have been detected, the insect can choose to follow a particular stimulus and thereby display host preference (this is what we have called *anthropophilic rate* i.e. the proportion of activated insects that chose human stimuli). Finally, when in the vicinity of the host, the insect reduces its flight velocity, lands, probes and eventually bites the host (i.e. short-range positive taxis) [52].

Why NA interfered with insect activation rate and not with orientation/host preference (two behavioural steps of the host-seeking process) remains enigmatic. A first possibility is that NA affects the physiological and molecular mechanisms responsible for olfactory transduction [57, 58], such that their ability to activate upon contact with host stimuli is inhibited. In other words, there might be variation in insect susceptibility to NA such that resistant individuals can complete the whole behavioural sequence of host-seeking, from activation to orientation and preference. When NA concentration increases, so does the proportion of susceptible individuals with inhibited olfactory/visual transduction pathways. Alternatively, rather than interfering with the molecular integration and regulation of olfaction (and vision for tsetse flies), NA may simply cause reduced general vigour. In this case, the stimuli sensing and transduction might work perfectly fine but the effectors (the muscles) do not respond. In any case, it will be important to study the behavioural effects of NA using field mosquitoes since rearing insects in the laboratory for many generations can cause the loss of certain genetic traits associated to host-seeking behaviour [59]. Here insects had been maintained on rabbits, for several generations before experimentation. Adaptation to feed on rabbits may have altered their responses to human and cattle feeding and confounded ability to pick up an impact of NA on anthropophily/feeding rates in the assay. Finally, we were able to study only one *Anopheles* and one tsetse fly species (i.e. *An. coluzzii* and *G. p. gambiensis*, respectively) and since there might be intra- and inter-specific variation in responses to NA, other mosquito and tsetse fly species/populations may respond differently. Our study shows that beyond causing reduced feeding on the mosquito host (i.e. the final step in the behavioural sequence of host feeding), NA can also interfere with vector response to host stimuli (the first step in the behavioural sequence of host feeding), here shown as decrease in the activation rate of *An coluzzii* at 2000 μg/ml and of *G. palpalis palpalis* at 12.5 μg/ml. From an epidemiological perspective, a decreased activation rate would lower the number of contacts with vertebrate hosts and hence reduce the probability of pathogen transmission. Future work should also explore the effect of neem administration to animal hosts on the attractiveness to the disease vectors. Do mosquito and tsetse vectors feed as readily on treated hosts (humans or cattle) as on untreated individuals? Is there a repellent effect of treated individuals on vectors? In particular, we do not yet know whether repellent effect of a compound added to a blood mixture in an artificial membrane feeder would translate to repellency when administered to a living host.

Future studies should mimic the real life situation in which vectors take blood meals every 2–4 days and address the impact of NA on the various vector fitness parameters after multiple treated blood meals. In such studies, mosquitoes and flies should be exposed to a range of NA dosages through blood meals on, e.g. treated mice, allowing the evaluation of bioavailability issues of the neem product and biological effects at the same time. From previous studies conducted with NA on the rodent malaria parasite *Plasmodium berghei* in BALB/c mice, it emerged that NA persisted in the plasma at a level able to affect parasite development for about 7 h [60]. A NA half-life of only few hours would require daily treatments (at least), a challenge not easy to meet. However, given the slower specific metabolic rate (the metabolic rate per unit mass) of large mammals in respect to mice, the half-life of azadirachtin may be longer in humans or cattle [61]. Clearly, further studies are needed to appropriately address NA pharmacokinetics issues in order to be able to fully evaluate the feasibility of the approach and eventually orient NA formulation strategies. For example in the case of cattle, the preparation of medicated feed may be the option of choice if a daily administration scheme is required.

Concerning toxicity issues, no adverse effects and no deaths were observed in any of the mice used in the *in vivo* acute toxicity experiment conducted in the frame of this study. Other acute toxicity investigations on NA showed a LD50 of > 3365 mg/kg in mice and of  > 5000 mg/kg in rats [62]. Subchronic oral toxicity experiments conducted in albino rats with azadirachtin technical (containing azadirachtin at 12 %) did not reveal any signs of toxicity after 90 days of administration at a daily dose of 1500 mg/kg in [63]. In any case, it will be fundamental to assess possible NA effects with chronic toxicity and teratogenicity protocols and study possible interactions of NA with other drugs and/or phytomedicines.

## Conclusions

Our findings indicate that in *An. coluzzii*, a major vector of *P. falciparum*, the ingestion of NA in bloodmeals, according to the dosage can result in (i) primary antifeedancy, (ii) decreased longevity, and (iii) reduced response to host odours. In *G. palpalis gambiensis*, a major vector of African animal and human trypanososmes, NA causes (i) knock-down effects, (ii) modified survival depending on the dose, and (iii) reduced response to host stimuli. In both cases, NA appears not to affect the anthropophilic rate of activated insects. Overall the most significant effects observed after a single treatment were the impact on mosquito survival at a NA of 2000 μg/ml and the knock-down of flies at 50 μg/ml. Further research is needed to evaluate field applicability of the approach. In particular, semi-field studies should be set up to test the effect of NA administered at various doses to bovine hosts on insect fitness parameters in mosquito and tsetse fly green-house facilities, allowing the bloodsucking insects to take up repeated blood-meals for a prolonged period.

## Additional file

Additional file 1: Figure S1. Photos of the feeding devices: **a** Mosquito membrane feeding assay, **b** Fly membrane feeding assay. **Figure S2. **Dual port olfactometer used for studying mosquito host-seeking behavior: **a** Photo of the indoor part of the device, **b **Photo of the out door part of the device, **c **Schematic representation of the dual-port olfactometer. **Figure S3. **Flight chamber used for studying fly host-seeking behavior: **a** Front photo of the device, **b** Schematic representation of the flight chambers. (PDF 450 kb)

## Abbreviation

NA: Neemazal ®
