# Supplementary material for: Neemazal ® as a possible alternative control tool for malaria and African trypanosomiasis?
Source: Parasit Vectors. 2016 May 4;9:263. doi: 10.1186/s13071-016-1538-x (PMC4857419; doi:10.1186/s13071-016-1538-x)
Supplement: Additional file 1: Figure S1. — Photos of the feeding devices: a Mosquito membrane feeding assay, b Fly membrane feeding assay. Figure S2. Dual port olfactometer used for studying mosquito host-seeking behavior: a Photo of the indoor part of the device, b Photo of the out door part of the device, c Schematic representation of the dual-port olfactometer. Figure S3. Flight chamber used for studying fly host-seeking behavior: a Front photo of the device, b Schematic representation of the flight chambers. (PDF 450 kb) [file 13071_2016_1538_MOESM1_ESM.pdf]

(a)

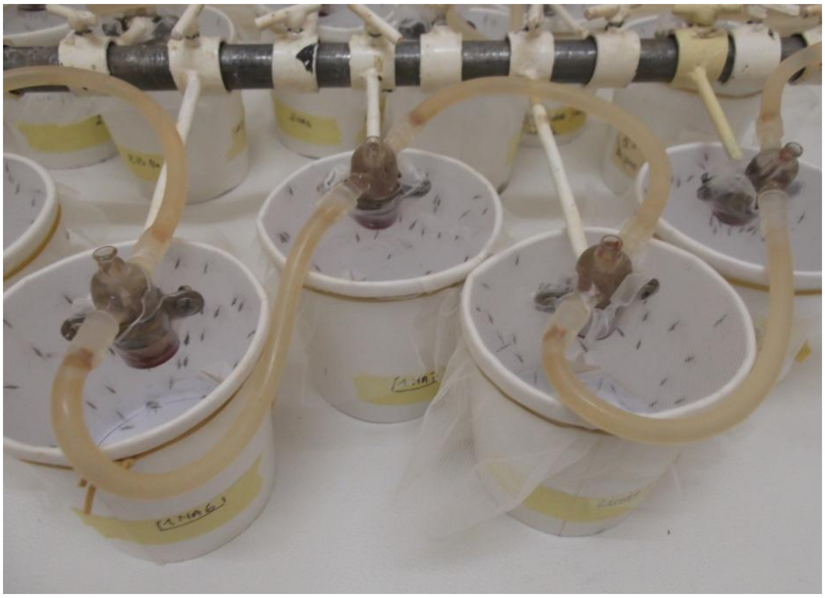

(b)

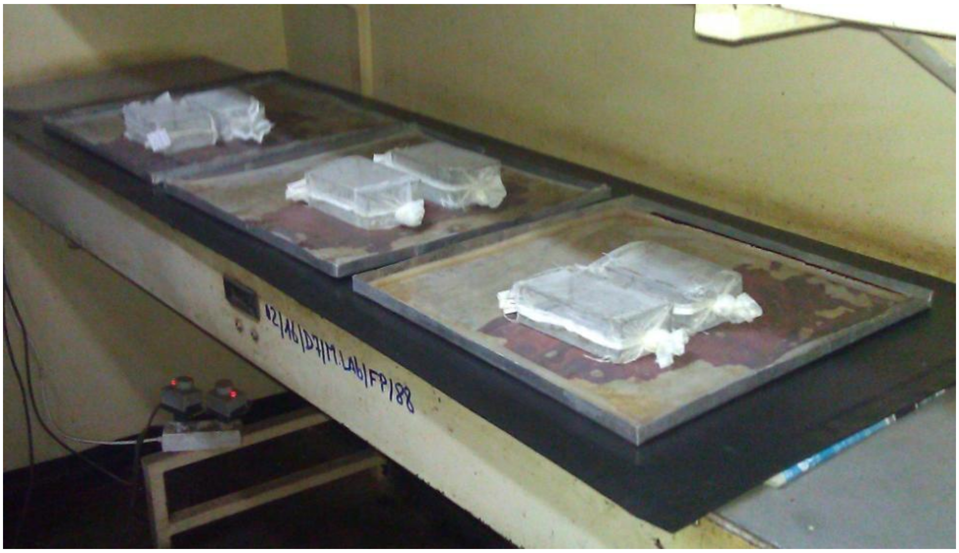

Figure S1. Photos of the feeding devices. (a) Mosquito membrane feeding assay (b) Fly membrane feeding assay

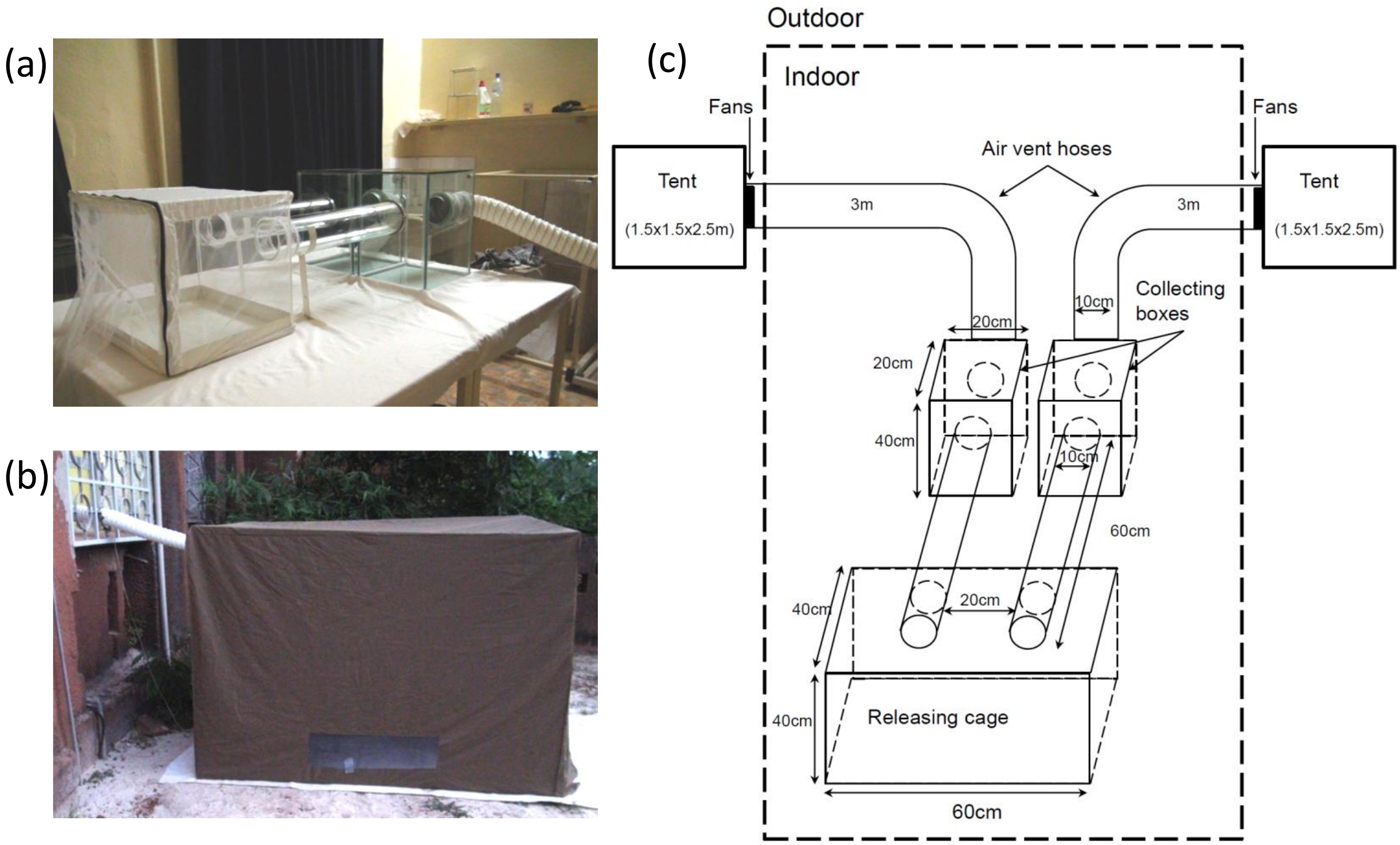

Figure S2. Dual port olfactometer used for studying mosquito host-seeking behavior. (a) Photo of the indoor part of the device, (b) Photo of the out door part of the device. (c) Schematic representation of the dual-port olfactometer.

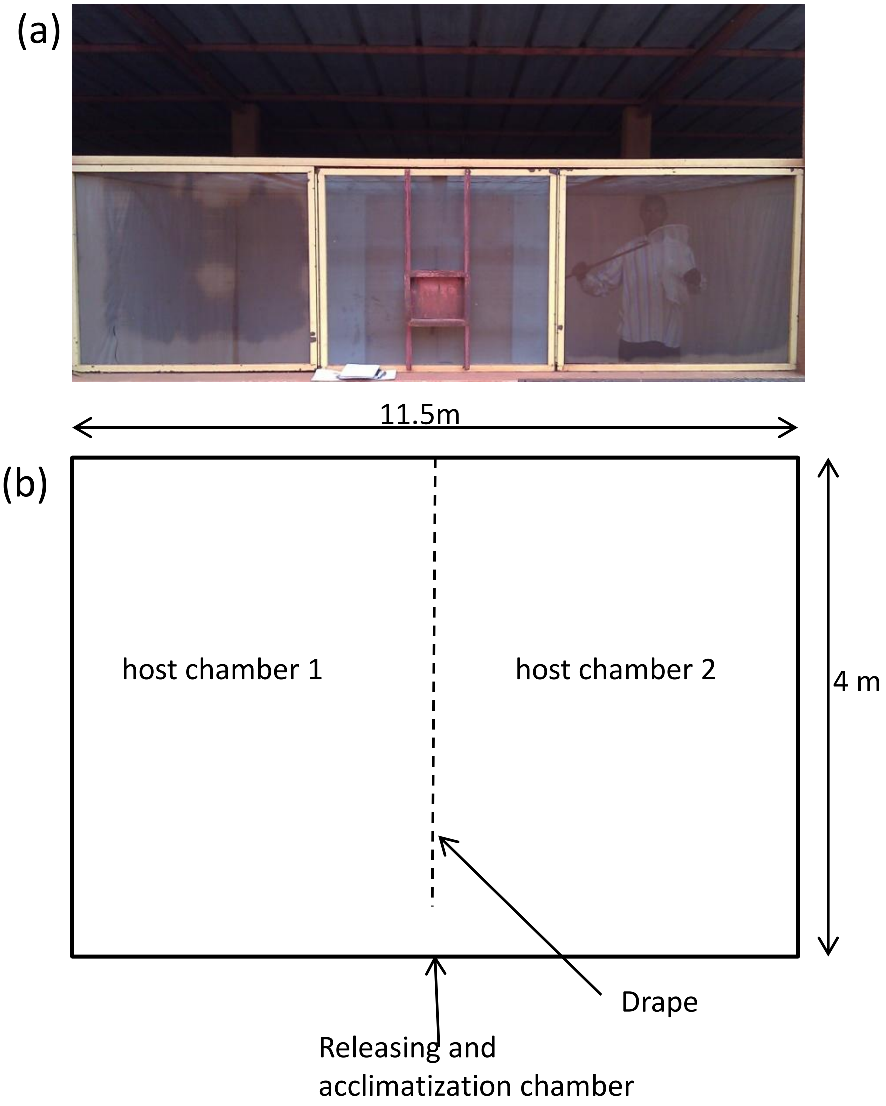

Figure S3. Flight chamber used for studying fly host-seeking behavior. (a) Front photo of the device, (b) (b) Schematic representation of the flight chambers.
